# Mild to moderate atopic dermatitis severity can be reliably assessed using smartphone‐photographs taken by the patient at home: A validation study

**DOI:** 10.1111/srt.13136

**Published:** 2022-01-12

**Authors:** Zarqa Ali, Andrei Chiriac, Theis Bjerre‐Christensen, Ari Pall Isberg, Priyanka Dahiya, Ionela Manole, Ana‐Maria Dutei, Irina Deaconescu, Adina Serban, Alina Suru, Tove Agner, Maria Rørbæk Kamstrup, Katrine Togsverd‐Bo, John Robert Zibert, Simon Francis Thomsen, Anders Daniel Andersen

**Affiliations:** ^1^ Department of Dermatology and Wound Healing Centre Bispebjerg Hospital Copenhagen Denmark; ^2^ OMHU A/S Copenhagen Denmark; ^3^ Studies&Me A/S Copenhagen Denmark; ^4^ Colentina Clinical Hospital Bucharest Romania; ^5^ Laurus Medical Pitesti Romania; ^6^ Dermestet Clinic Bucharest Romania; ^7^ Hebra Clinic Bucharest Romania; ^8^ Department of Biomedical Sciences University of Copenhagen Copenhagen Denmark

**Keywords:** agreement, assessment, atopic dermatitis, EASI, eczema, IGA, photograph, reliability, SCORAD, severity, severity, validity

## Abstract

**Background:**

The use of photographs to diagnose and monitor skin diseases is gaining ground.

**Objectives:**

To investigate the validity and reliability of photographic assessments of atopic dermatitis (AD) severity.

**Methods:**

AD severity was evaluated in the clinic by two assessors using the Eczema Area and Severity Index (EASI), SCOring Atopic Dermatitis (SCORAD), and Investigator's Global Assessment (IGA). Participants photographed the lesions with their own smartphone and completed a questionnaire about the extent of eczema the same day from home. The photographs were assessed twice with an 8 weeks interval by five dermatologists experienced in photographic evaluations. Intraclass correlation coefficients (ICC) with 95% confidence interval (CI) were applied.

**Results:**

Seventy‐nine participants were enrolled. The ICC between clinical EASI and photographic EASI was 0.88 (95% CI 0.81–0.93), and 0.86 (0.70–0.93) between clinical SCORAD and photographic SCORAD.

Perfect agreement between clinical IGA and photograph IGA was observed for 62%, with the difference between the two never deviating with more than 1 score.

The inter‐rater ICC for photographic EASI and photographic SCORAD, respectively, was 0.90 (0.85–0.94), and 0.96 (0.91–0.98). The intra‐rater agreements between the first and second assessments varied from 0.95 to 0.98 for photographic EASI, and from 0.86 to 0.94 for photographic SCORAD.

**Conclusion:**

There was high agreement between mild to moderate AD severity assessed clinically and based on smartphone photographs. Further, the photographic assessments can be reproduced with high reliability.

**Abbreviations**
AD: atopic dermatitis
APP: application
CI: confidence interval
DCT: decentralized clinical trials
EASI: Eczema Area and Severity Index
ICC: intraclass correlation coefficients
IGA: Investigator's Global Assessment
SCORAD: SCOring atopic dermatitis

## 1 INTRODUCTION

Atopic dermatitis (AD), is a chronic inflammatory skin disease,^1^ which affects up to 20% of children and 8% of the adult population.^2^ AD is characterized by eczematous infiltrated lesions with edema, vesicles, oozing, and crusting; and dominated by lichenification, excoriations, papules, and nodules in the subacute and chronic form.^3^

The evaluation and monitoring of AD severity rely on the assessment of clinical manifestations by a doctor along with subjective symptoms reported by the patient. There are no serological tests or other adequate laboratory tests to diagnose or categorize AD. It is extremely important that the assessment of AD severity is as objective and reproducible as possible. The use of photographs, especially taken by the patient with a smartphone, to diagnose and monitor AD and other skin conditions are gaining ground, and the evolution is being accelerated by the COVID‐19 pandemic. During the pandemic, to reduce the number of personal consultations and thereby to limit the spread of COVID‐19, the use of teledermatology has rapidly increased.^4^ The cornerstone of teledermatology is the evaluation of photographs of skin conditions captured by the patient. The reliability of photographs of skin conditions is not only relevant for clinical practice but also for clinical trials. Decentralized clinical trials (DCTs) are also gaining more attraction as they can accelerate patient recruitment, increase participant diversity, and bring medicines to market faster.^5^ With DCT the patients can participate in clinical trials from the comfort of their own homes with fewer, or even no in‐person clinic visits. Skin conditions can be monitored using photographs taken by the patient often with their own smartphones.^5^ Therefore, the validation of smartphone photographs to assess the severity of skin conditions is essential.

The objective of this study was to investigate the validity and reliability of photographic assessments of AD severity based on smartphone photographs taken by the patients from home, in combination with patient‐reported disease extent.

## 2 MATERIALS AND METHODS

This study examined the assessment of AD severity based on traditional clinical evaluation compared with the assessment based on smartphone photographs obtained by the patient at home, in combination with patient‐reported disease extent, in adults with AD. Participants with AD were recruited from the patient pool of already scheduled visits in the AD outpatient clinic at the Department of Dermatology, Bispebjerg Hospital, Copenhagen, Denmark, and from online recruitment through adverts on Facebook. Participants from the outpatient clinic had a confirmed AD diagnosis from a dermatologist, whereas participants from online recruitment were initially screened online using the UK Working Party Diagnostic criteria.^6^ Participants recruited online had the diagnosis confirmed by a dermatologist at the physical visit in the clinic.

On the day of examination in the clinic, AD severity was assessed twice by two clinical assessors using the Eczema Area and Severity Index (EASI),^7^, ^8^ SCOring Atopic Dermatitis (SCORAD),^2^ and Investigator's Global Assessment (IGA).^9^ The clinical assessors were blinded to each other's evaluations. The clinical assessments were performed by five dermatologists and one resident dermatologist that took turns. Qualified study staff then instructed the participant on how to take a good quality photograph with their smartphone, how to estimate the disease extent, helped the participant downloading the study photo application (app) onto the smartphone (Imagine, LEO Innovation Lab, Denmark), and provided instructions on how to navigate and use the app. Imagine is a user‐friendly app that supports patients photographing and monitoring their skin disease with all the necessary data security processes in place. In the clinic, the participants were encouraged to set a reminder in the app at a specific time later the same day to remind them of capturing the photographs at home.

On the same day of the examination, the participant was asked to take one photograph of a representative lesion in each of the four anatomical regions; face/neck, upper extremities, trunk, and lower extremities at their home. The lesions were selected by the first consulting doctor in the clinic and marked on a piece of paper handed out to the participant. Further, an online questionnaire regarding the extent of AD, and itch and sleep quality was completed from home. Itch and sleep were rated using a numerical rating scale ranging from “0” for no itch (or no sleeplessness) to “10” for the worst imaginable itch (or sleeplessness) from the SCORAD. The photographs taken by the participants were assessed by the clinical assessors and the photographic assessors twice with 8 weeks apart to explore the intra‐rater agreement.

### 2.1 Photographic severity assessments

SCORAD is a clinical scoring tool composed of both a subjective (itch and sleep quality) and an objective part consisting of both disease intensity and extent.^10^ The calculation of photographic EASI and photographic SCORAD depends on a combination of the patient‐reported extent, and the dermatologist's evaluation of the disease activity in the four body regions based on photographs taken by the patient. For each photo, the clinical signs known from SCORAD and EASI (erythema, edema/papulation, excoriations, lichenification, oozing/crusts, and dryness), along with their intensity (0–3) were rated by the dermatologists.

**FIGURE 1:**
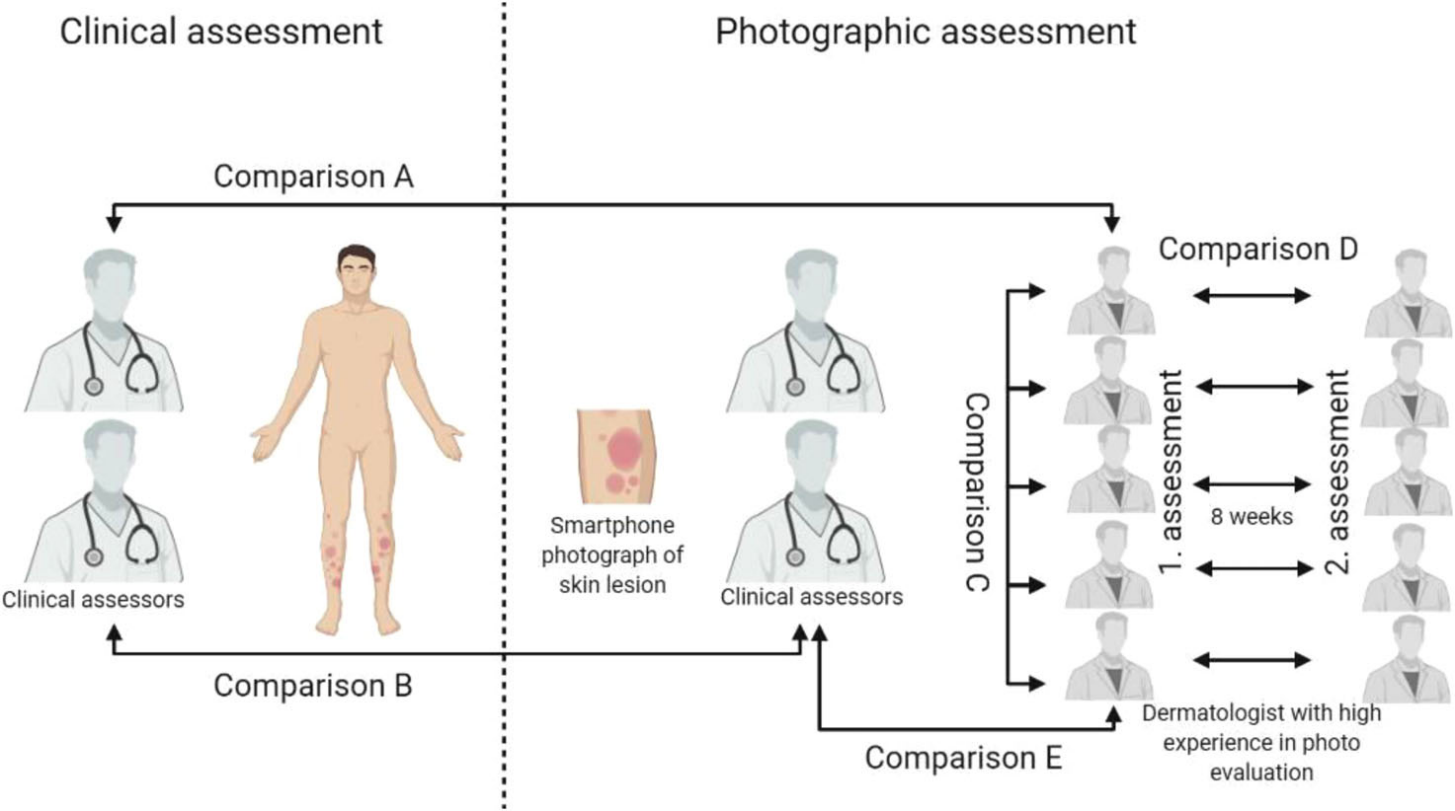
An overview of study design. Comparison (A): Inter‐rater reliability between clinical severity ratings and photographic severity ratings are done by five assessors experienced in photograph evaluations. Comparison (B): Inter‐rater reliability between clinical severity ratings and photographic severity ratings done by the same clinical assessor. Comparison (C): Inter‐rater reliability between dermatologists with high experience in photograph evaluations. Comparison (D): Intra‐rater reliability between first and second photographic severity ratings with 8 weeks interval by dermatologists experienced in photograph evaluations. Comparison (E): Inter‐rater reliability between photographic severity ratings done by clinical assessors and dermatologists experienced in photograph evaluations

All of the patient‐taken smartphone photographs were rated by the same clinical assessor completing the assessment in the clinic and also twice by a panel of five blinded board‐certified dermatologists (remote assessors). The remote assessors were highly experienced dermatologists with training in assessing photographs of skin diseases using secure browser‐based and purpose‐built dashboards on tablet and/or computer. To also investigate intra‐rater reliability, the photograph assessors rated each photo twice with at least 8 weeks in‐between. In addition, at least 8 weeks after the physical assessment in the clinic the photographs were evaluated by the same clinical assessor who had conducted the physical assessment in the clinic (Figure 1). Altogether this allowed for the calculation of measures of both validity and reliability of the photographic severity assessment method.

### 2.2 Statistical analysis

Pearson correlation and intraclass correlation coefficients (ICC) with 95% confidence intervals (CI) were calculated to evaluate the agreement between clinical and photographic assessments. In addition, ICC was also applied to investigate inter‐ and intra‐rater reliability of the photographic assessments.

The ICC estimates for inter‐rater agreements were based on a two‐way random‐effects model, absolute agreement, and average measure.^11^ For intra‐rater agreement it was based on the same parameters, except the use of a two‐way mixed‐effects model. An ICC >0.90, 0.75–0.90, 0.50–0.75, and <0.50 are generally agreed to indicate excellent, good, moderate, and poor agreement, respectively.^11^ For photographic EASI and photographic SCORAD the severity scores were calculated based on the dermatologist‐rated intensity from the photos combined with the participant‐reported extent (and subjective symptoms), as appropriate. For photographic IGA, an IGA score was assigned to each photo, and the maximum IGA per participant was then carried forward and averaged across the dermatologists. The percentage of perfect agreement was computed for the IGA. Statistical analyses were performed using the computing environment R (R Development Core Team, 2019) and RStudio (Boston, 2019). 

### 2.3 Ethical approval

The Danish regional Committee on Health Research Ethics was informed about the study, and because this was a method comparison study, the committee deemed that ethical approval was not required. The handling of patient‐sensitive data was approved by the Danish Data Protection Agency and compliant with General Data Protection Regulation.

## 3 RESULTS

A total of 101 participants (13 from the outpatient clinic and 88 from online recruitment) had a clinical visit scheduled. Twelve participants from the online recruitment did not pass the physical screening in the clinic as AD diagnosis could not be confirmed clinically, as some had only hand eczema or non‐atopic eczema. After the first clinical visit, six patients were excluded for not completing the online questionnaire from home, and four for not uploading the photographs, leaving 79 (78% women, 22% men) with a mean age of 35 (SD ± 15) years for final analysis. Based on the clinical EASI score, 20% (*n* = 16) had almost clear, 62% (*n* = 49) had mild, 13% (*n* = 10) had moderate, and 5% (*n* = 4) had severe AD.

### 3.1 Validity of EASI, SCORAD, and IGA assessed by remote assessors based on smartphone photographs

**FIGURE 2:**
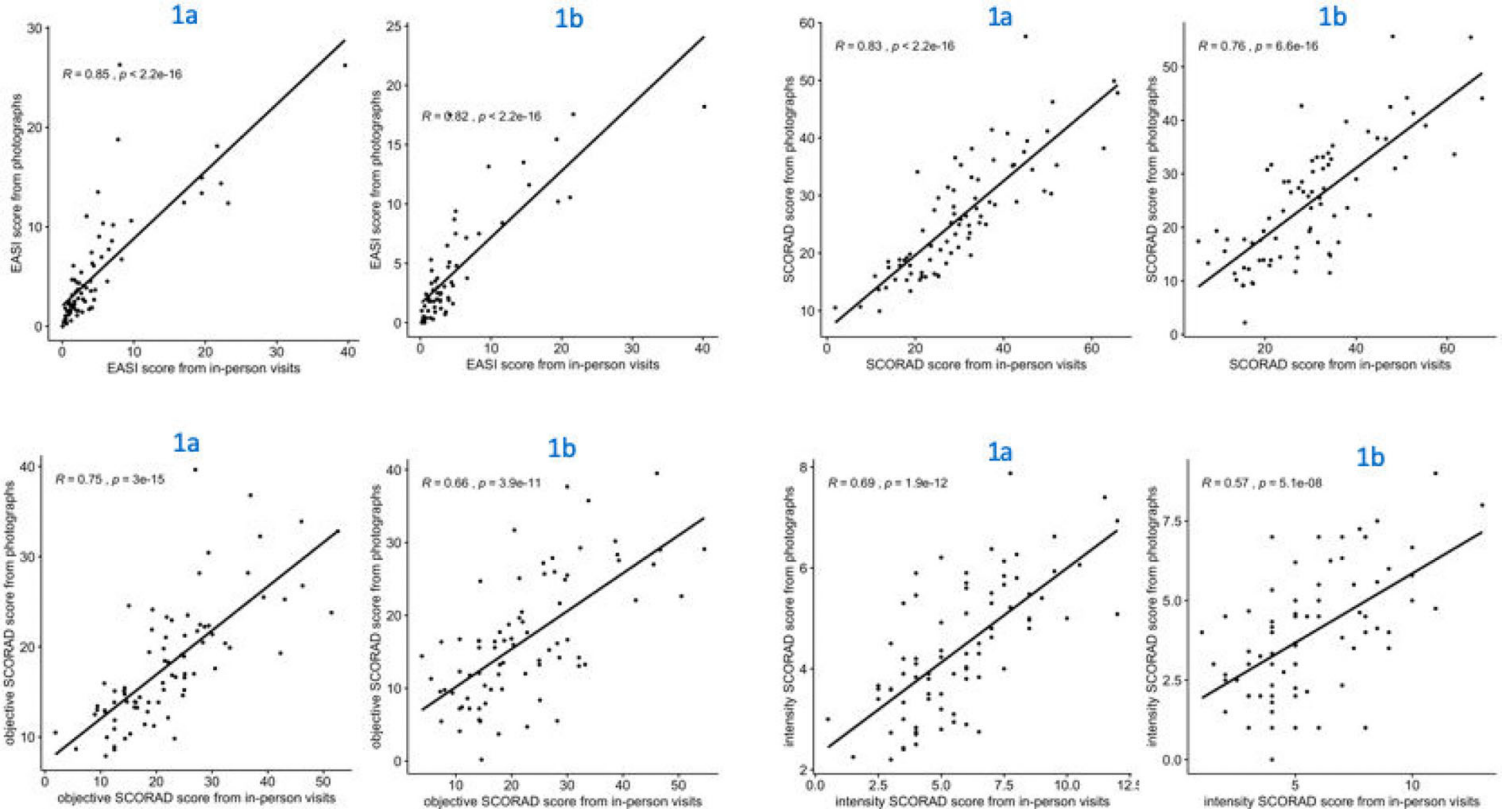
Correlations between clinical and photographic severity ratings of atopic dermatitis. 1a = correlation between clinical severity ratings and photographic severity ratings done by an assessor experienced in photograph evaluations. 1b = correlation between clinical severity ratings and photographic severity ratings done by the same clinical assessor

The correlations between clinical assessment and photographic assessment, when the photographic assessments were performed by five dermatologists with high experience in digital photograph evaluations (Figure 1, comparison A), were *r* = 0.86 (*p* < 0.0001) for EASI and *r* = 0.83 (*p* < 0.0001) for SCORAD (Figure 2). The ICC between clinical EASI and photographic EASI was 0.88 (0.81–0.93), and 0.86 (0.70–0.93) between clinical SCORAD and photographic SCORAD. For IGA, the perfect agreement between clinical and photographic evaluation was observed for 62%.

The ICC for inter‐rater reliability between the five dermatologists with high experience in photo evaluations (Figure 1, comparison C) was 0.90 (0.85–0.94) for photographic EASI and 0.96 (95% CI 0.91–0.98) for photographic SCORAD. For photographic IGA, perfect agreement was observed for 43%.

The ICC for intra‐rater reliability for first and second photographic assessments with 8 weeks interval (Figure 1, comparison D) varied from 0.95 to 0.98 for photographic EASI, and from 0.86 to 0.94 for photographic SCORAD. (Table 1)

**TABLE 1:** Intraclass correlation coefficients (ICC) and 95% confidence intervals for intra‐rater agreement between first and second photographic assessment done by the same rater

Remote raters	EASI	Total SCORAD	objSCORAD	iSCORAD	IGA
Rater 1	0.95 (0.91–0.97)	0.86 (0.74–0.92)	0.77 (0.59–0.86)	0.59 (0.37–0.74)	64%
Rater 2	0.97 (0.94–0.99)	0.90 (0.69–0.95)	0.79 (0.48–0.90)	0.58 (0.22–0.77)	76%
Rater 3	0.97 (0.94–0.98)	0.94 (0.90–0.96)	0.87 (0.79–0.91)	0.73 (0.61–0.82)	80%
Rater 4	0.98 (0.95–0.99)	0.89 (0.82–0.93)	0.79 (0.68–0.87)	0.64 (0.47–0.76)	78%
Rater 5	0.97 (0.93–0.99)	0.92 (0.86–0.95)	0.84 (0.74–0.91)	0.74 (0.57–0.84)	72%

Abbreviations: EASI, Eczema Area and Severity Index; IGA, Investigator's Global Assessment; iSCORAD, the intensity part of the SCORAD; objSCORAD, objective SCORAD; SCORAD, Scoring Atopic dermatitis.

### 3.2 Validity of EASI, SCORAD, and IGA assessed by clinical assessors based on smartphone photographs

The correlations between clinical severity ratings and photographic severity ratings, when the evaluations were done by the same clinical assessor (Figure 1, comparison B), were *r* = 0.81 (*p* < 0.0001) for EASI and *r* = 0.76 (*p* < 0.0001) for SCORAD. The ICC for EASI and SCORAD was 0.84 (0.73–0.91) and 0.82 (0.56–0.91), respectively. In addition, for IGA, the perfect agreement was observed for 47%.

### 3.3 Comparison between clinical assessors and remote assessors

The ICC for inter‐rater agreement for photographic severity assessments between clinical assessors and dermatologists with high experience in digital photo evaluations (Figure 1, comparison E) was 0.95 (0.90–0.97) for photographic EASI and 0.94 (0.89–0.96) for photographic SCORAD, respectively. For photographic IGA, perfect agreement was observed for 46%.

## 4 DISCUSSION

This study of 79 adults with AD showed good to excellent agreement between AD severity assessed by traditional clinical evaluation and based on smartphone photographs obtained by the patient at home in combination with patient‐reported extent, suggesting that AD severity can, with high validity be assessed digitally. Further, the photographic assessments had very low inter‐rater and intra‐rater variation, indicating consistency in photographic assessments and showing that photographic assessments are reliable.

Data from the present study demonstrates better intra‐rater and inter‐rater reliability of photographic EASI than photographic SCORAD and IGA. Similar results have been reported for traditional clinical assessments of AD. Zhao et al^12^ showed an excellent intra‐rater reliability of the EASI (0.886 (95% CI 0.744–0.952)) in a study with 12 patients and 5 dermatologists. However, the inter‐rater ICC (0.498 (95% CI 0.234–0.785)) and intra‐rater ICC (0.446 (95% CI 0.037–0.730)) was poor for objSCORAD. Bozek et al^13^ also demonstrated high intra‐rater reliability of the EASI (ICC 0.71) and moderate intra‐rater variability of the objSCORAD (ICC 0.66) and IGA (0.54) in a study of 10 patients assessed by 10 dermatologists. Further, the coefficient of variation (CV) for inter‐rater variability was high for EASI (CV 66.5) and moderate for objSCORAD (CV 28.1) and IGA (CV 33.0). In a study of 20 patients assessed by 15 dermatologists, Hanifin et al^7^ found the overall intra‐rater reliability of the EASI to be in the fair‐to‐good range. Wolkerstorfer et al^14^ showed an excellent inter‐rater agreement of the EASI (Cohen's kappa = 0.82; *p* < 0.001) in a study of 20 patients and three assessors. Hughes et al.^15^ in a reliability study, with 37 children and 33 adults, evaluated levels of agreement between assessments of AD in‐person and via digital photographs and found that AD can be reliably assessed via digital photographs using EASI, SCORAD, BSA, and IGA. However, these photographs were full‐body digital photographs captured by a clinical research coordinator in contrast to our present study where few photographs of representative AD lesions were taken by the patient at home. The latter is a more realistic scenario of how the photographs are used in clinical practice and will be used in DCT's. The results from the present study are in line with and adds to the knowledge on the reliability of AD severity assessment.

This study has some important strengths and limitations that need to be addressed. To our knowledge, this is one of the first studies designed to assess the validity and reliability of smartphone photographs taken by the patient at home to assess the severity of AD. The number of participants is high compared to previous studies and the assessment is completed by several assessors with different levels of experience. The main limitation is the lack of participants in the severe disease category. Further, extent of the disease reported by the patient is constant for agreement C, D, and E. This can result in a higher ICC for reliability for these comparisons. However, this is not the case for agreements A and B, and does not affect the agreement for IGA

In conclusion, this study showed excellent to good agreement between mild to moderate AD severity assessed clinically and photographically. Further, the photographic assessment had high reliability. This shows that patient‐obtained smartphone photos of representative AD lesions from each body region can be assessed with high consistency. The validation of smartphone photographs to assess the severity of skin conditions should be prioritized as it has already gained ground in clinical practice and clinical trials.

## CONFLICT OF INTEREST

ZA, SFT, KT, MRK, and TA have no conflict of interest. ADA, API, PD, IM, AD, ID, AS, AS, and TBC are employed by Studies&Me, and JRZ is CEO of Studies&Me.
